# Patient Involvement With Home-Based Exercise Programs: Can Connected Health Interventions Influence Adherence?

**DOI:** 10.2196/mhealth.8518

**Published:** 2018-03-01

**Authors:** Rob Argent, Ailish Daly, Brian Caulfield

**Affiliations:** ^1^ Beacon Hospital Dublin Ireland; ^2^ Insight Centre for Data Analytics University College Dublin Dublin Ireland; ^3^ School of Public Health, Physiotherapy and Sports Science University College Dublin Dublin Ireland

**Keywords:** patient compliance, rehabilitation, exercise therapy, biomedical technology, review

## Abstract

Adherence to home exercise in rehabilitation is a significant problem, with estimates of nonadherence as high as 50%, potentially having a detrimental effect on clinical outcomes. In this viewpoint, we discuss the many reasons why patients may not adhere to a prescribed exercise program and explore how connected health technologies have the ability to offer numerous interventions to enhance adherence; however, it is hard to judge the efficacy of these interventions without a robust measurement tool. We highlight how well-designed connected health technologies, such as the use of mobile devices, including mobile phones and tablets, as well as inertial measurement units, provide us with the opportunity to better support the patient and clinician, with a data-driven approach that incorporates features designed to increase adherence to exercise such as coaching, self-monitoring and education, as well as remotely monitor adherence rates more objectively.

## Introduction

The success of certain medical interventions depends largely on patient adherence to advice and prescribed rehabilitation regimes. After injury or surgery, many patients are given specific exercises to do unsupervised at home to aid their recovery, for example after knee replacement. These exercises are specifically targeted at certain muscle groups or joints, rather than global physical activity, for example a straight leg raise for quadriceps strength or a heel slide for knee range of movement. Evidence suggests that noncompliance to these home exercises in musculoskeletal cohorts can be between 30% and 50%, making it a significant issue that places additional burden on patients and health care providers, and may be partially to blame for poor clinical outcomes [1,2]. Clinicians who fail to consider patient adherence in rehabilitation programs may unnecessarily alter their treatment approach, face persistent patient complaints; or refer patients for alternative opinion, thereby contributing to possible unnecessary surgical intervention and additional health care costs [1]. Adherence is currently defined by the World Health Organization (WHO) as “the extent to which a person’s behaviour…corresponds with agreed recommendations from a health care provider” [3]. The majority of research into adherence relates to medication, and this definition has been developed to encompass a multitude of health-related behaviors [3], however, it does not yet include exercise prescription. In this viewpoint, we aim to comment on the current evidence in patient adherence to prescribed home exercise programs, the factors affecting these adherence rates, and then discuss design opportunities that connected health interventions provide to improve adherence rates.

The term *connected health* describes the use of a variety of technologies to inform and aid health care delivery in a data-driven manner with the individual at the center. It covers a broad domain, including digital, mobile health and telehealth and ensures all stakeholders are connected with data that is accurate and timely [4]. Mobile technology, including smartphones and tablets can support connected health solutions by providing access to a number of features within the device, including inertial measurement units (IMUs), cloud computing, and back-end development including machine-learning classification [5]. Equally, various methods such as telerehabilitation using videoconferencing [6], or motion capture using camera systems [7] have been employed with the aim to better support patients in their rehabilitation and improve their adherence. But what are the design features that can be harnessed in new connected health solutions that will impact adherence to exercise?

## Adherence to Exercise Programs

### An Operational Definition of Adherence in Exercise Science

Although medical adherence has been defined by the WHO, it is arguable that there is more than one factor to consider when specifically defining exercise adherence. To form a definition, the authors of this paper have considered on a macro level, the requirements needed to demonstrate strong adherence. Clinicians first need to know whether the patient is exercising, then whether they are exercising to the required amount of repetitions and sets, and finally whether they are performing the correct technique with relation to load, velocity, and alignment. The following operational definition has been built specifically relating to exercise, adapted from the WHO definition, and defines exercise adherence as “the extent to which an individual corresponds with the quantity and quality of exercise, as prescribed by their healthcare professional.” Those who perform their exercises to the required repetitions may not be deemed adherent should their technique be erroneous or incorrect, as these individuals will not be gaining maximum benefit from their exercise program.

### The Effect of Adherence on Outcome

Patient adherence is important in all aspects of medical care. Adherence is reported to have clear links to the impact on clinical outcome in medication research, as well as placing significant additional economic burdens on health care providers [8]. Poor medication adherence has been shown to increase the occurrence of hospitalizations and complications in a number of chronic metabolic conditions [9] and an increase in the number of adverse events and annual medical costs in cardiac patients [10].

In an exercise rehabilitation context among the musculoskeletal population, strong adherence enhances the effectiveness of the intervention and is suggested to reduce persistent, disabling complaints [11]. Patients who fail to adhere to the prescribed exercise program may extend the duration of their treatment, negatively impact on the therapeutic relationship, and make treatment less effective [12]. It can also impact health care providers with increased waiting times and poor efficiency [11], while poor adherence rates may also potentially have a role to play in nonsignificant outcomes of research papers [13]. A number of studies have also linked strong exercise adherence to improved treatment outcome in patients experiencing neck and back pain and osteoarthritis symptoms [14-16].

### Rates of Adherence

In an exercise context, it was reported that only 35.0% (42/120) of patients were highly adherent with home exercises in a short-term study of patients with nonspecific low back pain [17]. This study went on to state that 50.8% (61/120) of those who received an individualized exercise program demonstrated non/low adherence across the entire rehabilitation regime based on patient self-report. Yet the literature demonstrates some inconsistency in the use of the term non/low adherence, and there is no clearly documented category to which adherence can be defined as poor [18]. A more recent systematic review of interventions designed to improve adherence in a variety of musculoskeletal and medical populations found an average rate of 67% (12 studies) adherence to prescribed home exercise programs [19]. A regularly cited study by Sluijs et al [2] concluded that patients’ compliance to physiotherapy is unsatisfactory, but was unwilling to draw a sound conclusion on the degree of nonadherence due to the lack of valid and reliable measures available. From these findings, it is clear that adherence rates to home exercise plans are an issue, but it is not possible to accurately say to what extent, and how much this might impact the clinical outcome, as a consistently valid and reliable method of measurement has not yet been designed.

### Measuring Adherence

It is important to consider that studies assessing adherence are limited in their quality and conclusions because of the lack of objective and reliable outcome measures used in clinical practice. It is widely accepted that at present, there is no gold standard for the measurement of adherence to unsupervised home-based exercise, as the significant proportion of outcome measures used in the literature rely on patient self-report and are therefore susceptible to bias [19,20]. In a systematic review of 61 different self-reported outcome measures for adherence to home-based rehabilitation, only two measures scored positively for a single psychometric property of validation [20]. Furthermore, the outcome of any research studies using paper diaries or retrospective recall has been called into question as it is highly prone to recall and self-serving bias [21]. Equally, these measures make no allowance for the quality of performance, as highlighted in the abovementioned definition.

Sensing platforms such as the use of IMUs or motion capture camera are rapidly advancing and could be an opportunity to make a more objective assessment of adherence, continuously tracking motion data obtained from an individual [22,23]. However, the use of these devices to measure adherence is questionable as they arguably influence/enhance adherence itself by means of the user knowing that they are being recorded. In this way the end point is influenced greatly by the measurement strategy, leading to questionable results as the patient no longer has the choice on whether to adhere [20]. Regardless of the challenges with accurately measuring adherence, it is clear that there are problems with adherence to prescribed exercise in the home setting. Investigations of whether technology can play a role in this are still in their infancy, therefore, understanding what factors affect adherence can highlight design considerations a connected health solution can employ to improve adherence and aid self-management.

## Factors Affecting Adherence

### Overview

The factors that may affect adherence to home exercise rehabilitation in musculoskeletal populations have been discussed in numerous papers, and a number of characteristics have been highlighted as potential reasons that may affect or predict adherence rates [24] such as perceived barriers, the patient’s own beliefs, or their self-efficacy with the exercise task. Good adherence requires the individual to change, alter, or even maintain a behavior, hence it is relevant to consider the psychological factors associated with theories of behavior change, as guidelines suggest these should all have a theoretical underpinning [25]. While there are numerous theories of behavior change [26], very few physiotherapy studies (12%, 3/25) discuss these theories [27]. While behavior change is inherently included within the factors affecting adherence, and indeed within the design solutions offered, given the expansive nature of behavior change, the broad factors and barriers to adherence will be addressed in this paper.

### Self-Efficacy

Self-efficacy has been strongly linked as a psychological factor affecting treatment adherence. It is a term used to describe an individual’s belief in their own capability to achieve a task that will produce a targeted result. It is situation-specific and depends on the activity, but it is considered that a person has a general level of self-efficacy across tasks [28]. Four strands of efficacy information are proposed within the concept; mastery experiences based on past and current successful performance, social observation learning from those around the individual, persuasive information particularly from influential people in the individual’s life, and emotional states considering the mood the individual is in [29]. Self-efficacy has been closely linked with a positive association of adherence in orthopedic and musculoskeletal cohorts [24,30,31]. It is worth noting however, that one study found that self-efficacy did not predict adherence in the home or clinic setting, although this was assessed in a sports rehabilitation context, and therefore may not be generalized to other cohorts [32]. When designing connected health solutions, there is an opportunity to use interventions to improve self-efficacy within the technology design. Methods such as machine learning with biofeedback, interactive education using videos and weblinks, and self-monitoring similar to that used in commercially available fitness trackers, have the potential to improve the self-efficacy and ultimately the adherence of users.

### Threat and Beliefs

The beliefs a patient holds regarding their condition are also said to be a direct factor affecting adherence, and the decisions made by patients are based on their own beliefs, personal experiences, and the information they receive [33]. This study noted that those who did not perceive their injury to be serious demonstrated lower levels of adherence, and in fact, the authors suggested that enhancing participants’ level of threat to further injury or disability would improve adherence, although this is a questionable technique in the wider context of patient management. Indeed, others have stated that providing too much information to patients and overloading them will also negatively affect adherence, as patients can become confused [2]. Enhanced threat can also have other negative implications, such as hemophiliac patients who may have had a threat of increased bleeding and arthropathy with physical activity [34], and therefore treatment should be about correcting falsely construed beliefs and tailoring individual care, rather than solely modifying the threat appraisal for all patients. This individualized care is an important consideration in the design of connected health solutions, as the end user needs to be considered and technology should be used to augment the clinician’s management, rather than to replace in a *one size fits all* approach that may incorrectly adjust a user’s beliefs. Symptoms also need to be perceived to have a sufficient effect on quality of life to encourage adherence [35], with another viewpoint that the beliefs a patient holds places them in a similar category as consumers, who want to take their own decisions when confronted with a particular condition [8].

### Locus of Control

A recent systematic review into factors affecting adherence in low back pain suggested that a higher health locus of control had moderate evidence to be a factor affecting adherence [36]. Locus of control can be biased toward either the internal (person is responsible for their own outcomes), to chance, or to powerful others (individuals of higher authority are responsible for outcome) [37]. It is suggested that patients with an external locus of control demonstrate a lesser degree of adherence with medical intervention [2]. Hence, as a clinician it is imperative that both the patient’s beliefs and understanding of their locus of control are addressed at an early stage when considering a connected health solution to ensure the patient understands the condition and that possible misinformed beliefs can be corrected.

### Pain

Pain levels during exercise in musculoskeletal patients presented strong evidence as a barrier to adherence in a systematic review, but there was conflicting evidence that higher pain levels at baseline had an effect on adherence [24]. The authors suggested that those who experienced pain during their exercises were less likely to adhere to their program. Contradictory to this is the large study from the Netherlands that found there was no significant difference in reported pain from exercise between those with high and low adherence [2]. Brewer et al on the other hand, make links between pessimism and pain, with patients low in pessimism completing the exercises irrelevant of pain, while highly pessimistic individuals demonstrated a reduction in adherence when their pain levels were higher following cruciate ligament reconstruction [38]. Mobile technology has the ability to capture pain scores with a method requiring little interference in the user’s life, and when combined with the ability to objectively monitor adherence, may provide greater levels of understanding on the relationship between pain and adherence in future research. Connected health solutions may also be used to change the way care is provided, with the user completing regular Web-based outcome scores, and an increase in pain flagged to the clinician remotely, giving the health care professional an opportunity to make an informed decision on that patient’s care to ensure they maintain strong adherence.

### Physical Activity

The level of physical activity of individuals at their baseline has also been discussed as a potential barrier to adherence. Studies suggest that those who are physically active at baseline demonstrate significantly better adherence to home exercise programs [16,24]. Physical activity is also said to be a source of self-identity, and that individuals who have lower athletic identity would have a lower rate of adherence [38]. Connected health solutions have the opportunity to encourage physical activity through self-monitoring and gamification which could then, in-turn, contribute to the behavior change required for stronger adherence in rehabilitation. However, given that baseline physical activity cannot be altered by commencing use of a connected health solution, the design of the intervention needs to be future present and independent of the user’s baseline physical activity.

### Psychological Symptoms

Depression as a barrier to adherence has strong supporting evidence [24], with the literature also discussing other traits including anxiety and neuroticism. These symptoms have been suggested to negatively impact adherence in general musculoskeletal and fibromyalgia populations [38-40]. More recently, a study of cruciate ligament reconstruction participants found no link between anxiety at baseline and adherence [38], although interestingly it went on to suggest that day-to-day variance in stress may contribute to adherence to home exercises. If connected health technology can be used to either counteract these symptoms through recognized support methods or be able to flag to the clinician that adherence has dropped; this can lead to a more proactive method of health care to identify the reasons for this reduction.

### Social Support

The social support network of the patient has also been suggested as a possible factor in adherence [2,41]. This network can be friends and family members, as well as support from the therapist. In the sporting population, significant findings were made that both social support as task appreciation, and emotional support from friends and family predict adherence in both the clinic and home setting [32]. Further exploratory work in the sporting population made suggestions that those who made use of social support displayed greater adherence and recommended that in this setting, the coach and wider support network are involved in the rehabilitation pathway to offer support and motivation [42]. Connected health solutions have the potential to offer social support through online forums or networks where users are able to interact with others in a similar situation, wherever they may be. It is not impossible to foresee a social network built into many mobile health technologies to enhance patient experience and improve adherence. A recently published systematic review found relatively strong evidence that social support can predict adherence, but as discussed earlier, highlighted the challenge of measurement of adherence as a significant limitation across the field [18].

### Perceived Barriers

Patients’ perceived barriers is one of the most widely documented barriers to adherence, with examples such as forgetting to exercise, not having the time, or not fitting into the daily routine all being cited as reasons for nonadherence [2,42-44]. Another study also found perceived barriers included time, work schedules, and transportation and recommended that these issues should be taken into consideration by health care providers [17]. By using a selection of the design considerations discussed below, connected health solutions have the potential to positively influence some of these perceived barriers.

## Design Considerations for Connected Health Solutions

### Overview

When designing future connected health solutions, it is important to have an understanding of the range of possible cognitive, behavioral, and practical barriers that can have an effect on a patient’s willingness to adhere to their program [45]. The use of mobile devices connected with a form of sensing platform (camera or IMU) in home-based exercise rehabilitation have the potential to provide the clinician with a greater amount of actionable data, which will assist in the management of each case and shift to a more proactive approach to health care. By understanding the factors discussed previously, it is possible to build features and interventions in to new solutions with the aim of enhancing patient adherence and ultimately, clinical outcome.

### Coaching

By incorporating regularly combined strategies of supervision, feedback, and reinforcement as a design consideration, it is possible to offer the greater coaching input that a patient receives in clinic with their health care professional but in the convenience of their home environment. When physiotherapists provide positive feedback, and monitor both performance of exercises and the progression of symptoms, adherence rates have been found to be higher [2]. The design of connected health interventions can then offer supervision in the form of remote monitoring via online cloud-based portals. This coaching system can be augmented with remote communication using platforms such as videocalling, instant messaging, or email to offer further coaching components. Telerehabilitation has been extensively researched by a Canadian group who in one study of postoperative knee replacement patients found telerehabilitation in the form of videoconferencing to be as effective as usual care and had the potential to increase access to services [6].

Incorporating real-time exercise coaching into a connected health technology is a challenge, but research is ongoing to establish the feasibility of this process using an IMU to measure and classify commonly prescribed home-based exercises [5]. Bassett discussed how feedback from exercise testing can increase adherence in home-based exercises, as patients who know they are performing the task correctly are more likely to adhere, and results of the testing will increase self-efficacy [46]. One study of athletes after sports injury found that patients reported regular coaching was useful to aid adherence for two reasons: improving on exercise technique and also to act as a reinforcement to complete the exercises [42]. Technology can potentially offer this coaching in more visually stimulating ways than previously imagined, with audio reinforcement during use and 3D modeling using an avatar with input from devices including cameras or sensors. For example, the VERA system by Reflexion Health utilizes the Microsoft Kinect Camera to feed in to an avatar on the laptop or television screen which mirrors the user’s movements and guides them through an exercise program [7].

Task appreciation, when patients are complimented for their achievements, particularly for adherence [47], is particularly applicable in the gamification of health interventions to further compliment a user’s achievements. Whether this is via rankings, rewards, in-exercise games, or simply augmenting the experience with an avatar type feedback, the user can enjoy a more immersive experience within a connected health technology, potentially impacting on their adherence.

### Goal Setting

Goal-setting is regularly used to motivate and encourage adherence in physiotherapy, yet the literature seems to offer conflicting evidence on its effectiveness. Bassett and Petrie found no significant difference in adherence when comparing the use of goal setting [48]. This paper concluded that goal setting may not be a suitable motivational tool in patients with lower limb injury, although it did note that collaboratively set goals appear to have a higher level of adherence than those dictated from the therapist, although the issues with measuring this using diaries has been discussed earlier. A more recent study supported goals in clinic-based rehabilitation alongside other adherence improving interventions [32], and goals that were set with the support of a psychologist found significant differences in adherence in a moderate quality study of a younger athletic population [49]. A systematic review recently performed, concluded that while goal setting may be effective, there was insufficient data to make an endorsement, and more specialized skills may be required for goals and goal setting to be effective [19]. Arguably by making a prescription of exercise, a physiotherapist is already setting a goal for their patient, and therefore measuring the effect of formal goals is more difficult.

### Self-Monitoring

A number of studies use self-monitoring as a form of measurement, yet this in itself could be considered an adherence facilitator [21]. Activity monitors have been used to provide visual feedback to patients on their physical activity and exercise frequency. This intervention was found to have a positive association to adherence, when compared with a control group with the same monitor but without feedback. However, this was not in the musculoskeletal population and was targeted at general physical activity rather than targeted home exercises [34]. Talbot et al [50] also undertook a randomized trial using an accelerometer to allow for self-monitoring as part of an arthritis self-management program and found a notable increase in general physical activity. Self-monitoring with the use of IMUs therefore provides a method of reliable, objective self-monitoring, taking the concepts from the extremely successful fitness sector and applying them to health care in connected health applications.

### Education

Education is also an intervention to improve adherence; it is multifactorial and can affect perceived barriers and the patients’ beliefs/perceived threats that are discussed above. Studies using solely education are few and far between, but in a systematic review, no statistically significant findings were made on 2 fair quality studies, but the provision of written information in supplement to verbal instruction did improve adherence compared with verbal instruction alone [19]. A recent symposium piece also concluded that patients rarely need just more education, they need assistance with behavior change in an integrated program [51], perhaps suggesting that clinicians should be more aware of the psychological theories discussed previously. Whether Web-, tablet-, or mobile phone-based, connected health solutions can easily offer educational material in a variety of formats, including more interactive methods such as videos that would not have been available in the past to patients.

The majority of these interventions have been combined to form a self-management plan, and this is widely done in clinical practice. Evidence would support the use of varying strategies, targeting patient education and behavior modification and would be suggested as the most effective method of improving adherence provided it is tailored to each individual’s needs [52]. Although specific to the arthritis population, a systematic review concluded that at the time that there was limited evidence for adherence interventions for exercise, though adherence was not the primary outcome for some studies included [53]. Furthermore, although insufficient evidence was noted, Peek et al [19] concluded that pending further research, written information should be integrated into routine practice to enhance adherence, and this is easily provided in both written and video format in connected health solutions. Supporting the potential connected health opportunities, they suggested again with support of future research that activity monitors in the form of IMUs could be effective and simple to use to promote and monitor adherence. They also noted that this type of strategy would be increasingly acceptable as the population becomes more skilled with technology.

Other simple features that can be incorporated into the design for future solutions include automatic reminders with consideration for the patient’s daily routine [42], regular patient reported outcome measures specific to the target population to provide more meaningful data back to the clinician, and social forums that allow the user to interact with peers and share experiences, offering the social support discussed earlier. A number of these features were discussed by patients when interviewed regarding their expectations of new technologies in this area, with feedback to improve performance and encouraging a feeling of being more supported being recurring themes [54]. A recent parallel-group trial suggested that app-based exercise programs with remote support can improve adherence in exercise rehabilitation based on self-report, compared with paper handouts [55], but arguably, there is more that technology can offer to further improve and measure this facet of rehabilitation. Bassett sums up the objective well, when stating that prevention of nonadherence is the ideal way of maximizing adherence [1], and using connected health to move toward a more proactive model of care achieves this.

## Conclusions

Adherence to home exercises in rehabilitation is a significant problem, and the reasons for this are multifactorial, covering both psychological and situational factors that vary between each individual, and that need to be considered by clinicians in the design of personalized exercise programs. Techniques discussed in this paper can be built into connected health solutions with the aim to improve self-efficacy and ensure the patient feels better supported in their rehabilitation; this may have an effect on adherence rates and will provide clinicians with more meaningful data to base their clinical decision on. Furthermore, published research needs to investigate the impact of these solutions on adherence rates, as this is sparse at present, yet this is understandable given the difficulties in measuring adherence discussed within the paper.

Connected health technology has the potential to make an impact in the way we manage health and can provide a platform for a far more proactive method of management utilizing numerous interventions to further improve adherence, and ultimately rehabilitation outcomes for patients. There is an emerging market in the use of sensing systems to support patients in their rehabilitation, particularly around adherence to home exercise, although the published research is still in its infancy. These systems have the ability to include many of the design features discussed in this viewpoint within the developed system and have the ability to utilize ubiquitous and cost-effective hardware in the form of mobile phones and tablets. It may then also be possible for these systems to provide a more objective method of measuring adherence across clinical populations.

## Abbreviations

IMU: inertial measurement unit
WHO: World Health Organization
